# Supplementary material for: Interaction between Cervical Microbiota and Host Gene Regulation in Caesarean Section Scar Diverticulum
Source: Microbiol Spectr. 2022 Jul 28;10(4):e01676-22. doi: 10.1128/spectrum.01676-22 (PMC9430964; doi:10.1128/spectrum.01676-22)
Supplement: Supplemental file 1 — Supplemental material. Download spectrum.01676-22-s0001.pdf, PDF file, 1.2 MB [file spectrum.01676-22-s0001.pdf]

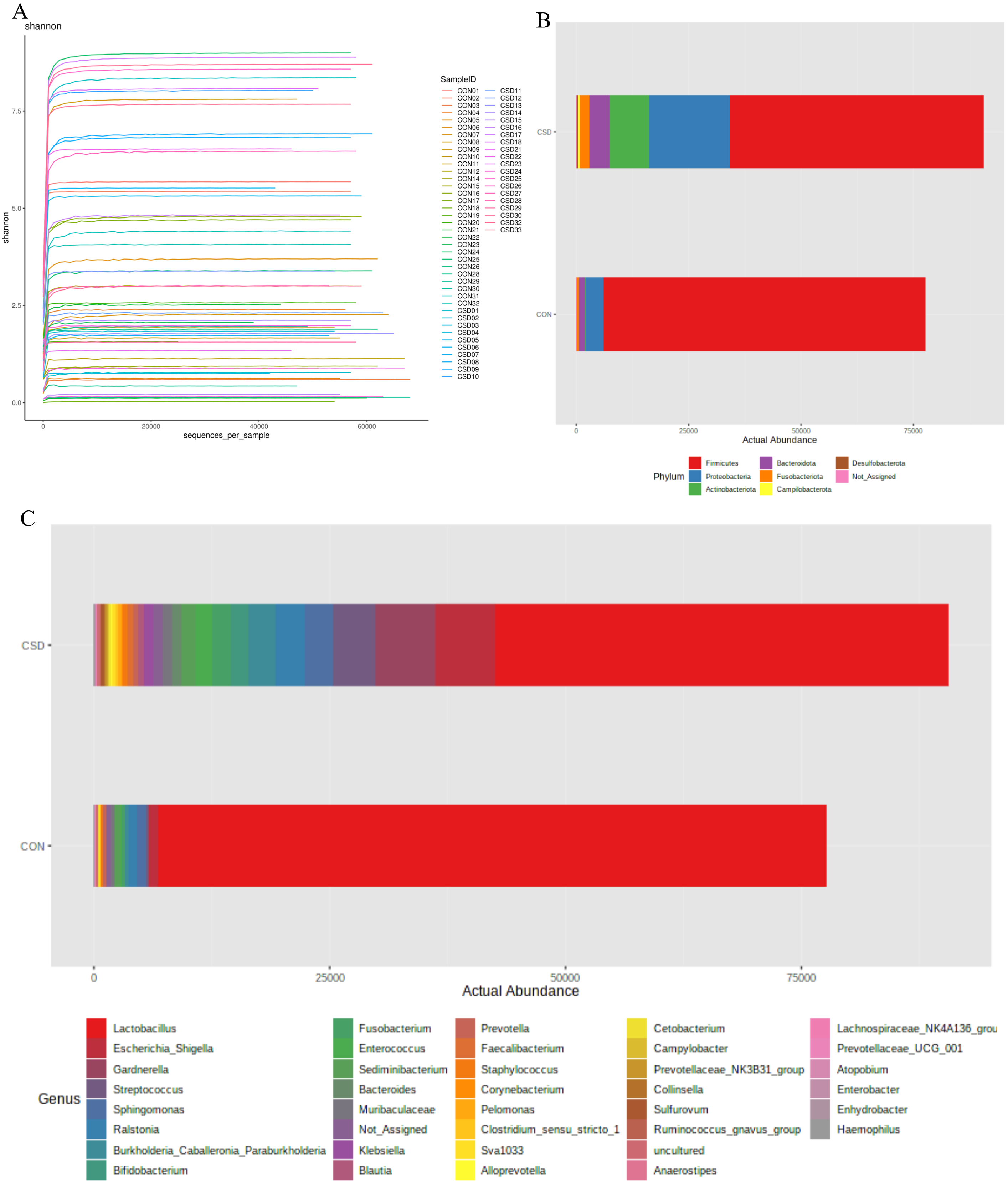

Supplementary Figure 1 (A). Rarefaction curves for each sample; Stacked graph of absolute abundance of CSD group and CON group at the phylum (B) and genus (C) levels.

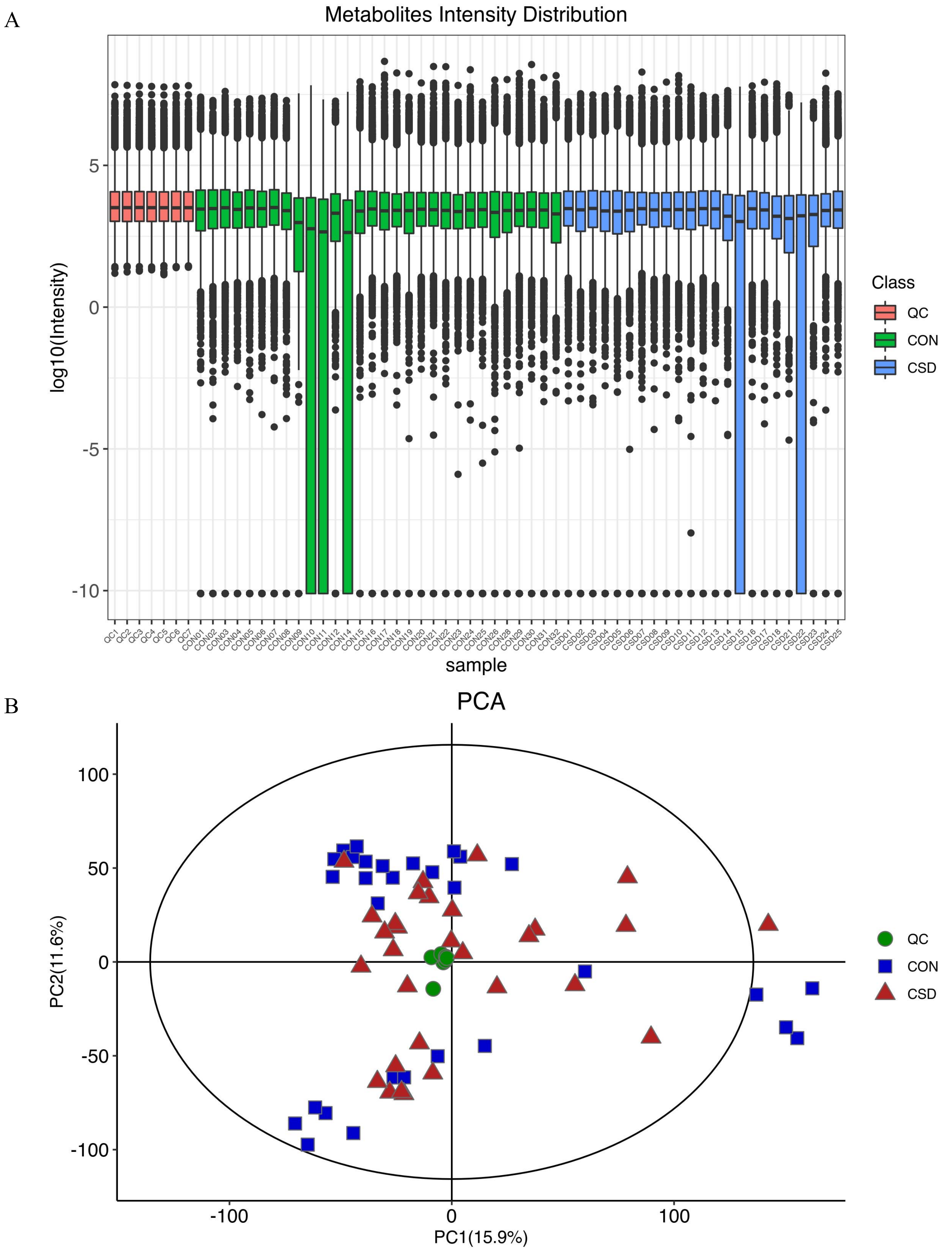

Supplementary Figure 2 (A). Metabolite intensity distribution boxplot; (B) PCA plots of two groups of metabolites and quality control samples.
